# Computer-Assisted Discovery of Alkaloids with Schistosomicidal Activity

**DOI:** 10.3390/cimb44010028

**Published:** 2022-01-15

**Authors:** Renata Priscila Barros de Menezes, Jéssika de Oliveira Viana, Eugene Muratov, Luciana Scotti, Marcus Tullius Scotti

**Affiliations:** 1 Post-Graduate Program in Natural Synthetic Bioactive Products, Federal University of Paraiba, João Pessoa 58051-900, PB, Brazil; renatapcbarros@gmail.com (R.P.B.d.M.); viana_jess@hotmail.com (J.d.O.V.); luciana.scotti@gmail.com (L.S.); 2 Laboratory for Molecular Modeling, Division of Chemical Biology and Medicinal Chemistry, UNC Eshelman School of Pharmacy, University of North Carolina, Chapel Hill, NC 27599, USA; murik@email.unc.edu

**Keywords:** *Schistosoma mansoni*, random forest, MuDRA, molecular docking, alkaloids

## Abstract

Schistosomiasis is a chronic parasitic disease caused by trematodes of the genus *Schistosoma*; it is commonly caused by *Schistosoma mansoni*, which is transmitted by *Bioamphalaria* snails. Studies show that more than 200 million people are infected and that more than 90% of them live in Africa. Treatment with praziquantel has the best cost–benefit result on the market. However, hypersensitivity, allergy, and drug resistance are frequently presented after administration. From this perspective, ligand-based and structure-based virtual screening (VS) techniques were combined to select potentially active alkaloids against *S. mansoni* from an internal dataset (SistematX). A set of molecules with known activity against *S. mansoni* was selected from the ChEMBL database to create two different models with accuracy greater than 84%, enabling ligand-based VS of the alkaloid bank. Subsequently, structure-based VS was performed through molecular docking using four targets of the parasite. Finally, five consensus hits (i.e., five alkaloids with schistosomicidal potential), were selected. In addition, in silico evaluations of the metabolism, toxicity, and drug-like profile of these five selected alkaloids were carried out. Two of them, namely, 11,12-methylethylenedioxypropoxy and methyl-3-oxo-12-methoxy-n(1)-decarbomethoxy-14,15-didehydrochanofruticosinate, had plausible toxicity, metabolomics, and toxicity profiles. These two alkaloids could serve as starting points for the development of new schistosomicidal compounds based on natural products.

## 1. Introduction

Neglected tropical diseases (NTDs) are a global public health concern. Most prevalent in Latin America, Africa, and Southeast Asia, NTDs generally affect the poorest populations, which are living in lower sanitary and socioeconomic conditions. According to the World Health Organization, there are 17 major parasitic infections, of which, the neglected tropical diseases are schistosomiasis, leishmaniasis, trachoma, lymphatic filariasis, and Chagas diseases [1,2,3].

Schistosomiasis is a chronic parasitic disease caused by trematodes of the genus *Schistosoma*, where the most common causes of the disease are *Schistosoma mansoni* and *Schistosoma haematobium*, which are transmitted by *Bioamphalaria* and *Bulinus* snails, respectively. Studies show more than 200 million people are infected with *Schistosoma* and that more than 90% of them live in Africa [4,5].

Pyrazinoisoquinoline derivatives with anthelmintic activity, such as praziquantel, were discovered in 1972 [6,7]. To date, numerous studies have suggested praziquantel to be safe, efficacious, and cost-effective [8]. However, hypersensitivity and allergic reactions, as well as drug resistance in *Schistosoma*, are frequently presented after administration [9]. Despite this, praziquantel remains the only clinical treatment for schistosomiasis, highlighting the need to investigate and develop new therapeutic agents with potential schistosomicidal activity.

Recent publications demonstrated that more than 50% of the new drugs approved worldwide are derived from natural products, showing an important role of secondary metabolites for identifying new compounds with potential therapeutic activity and low toxicity [10,11,12]. In fact, there are recent studies based on secondary metabolites that have shown that alkaloids have potential against *S. mansoni* [13], which appears to be a promising starting place for drug discovery.

In the last few years, modern drug discovery has utilized structure- and ligand-based computer-aided drug design (CADD) to identify promising new chemical compounds. Allied to this, the virtual screening (VS) method is a valuable tool in theoretical simulation, calculation, and prediction, and could guide and assist in discovering new small compounds, which shortens the time for designing new drugs and reduces the costs of drug development [14,15].

Studies with VS have helped to identify new compounds with in vitro assays using a virtual screening of a compound library, which demonstrates the computational efficiency in identifying new compounds [14,15]. Therefore, in our study, a combination of both structure- and ligand-based screening techniques were used to select the best representatives of drugs derived from Menispermaceae and Apocynaceae families active against *S. mansoni.*

## 2. Materials and Methods

### 2.1. Dataset

Four sets of chemical structures with known activities against *S. mansoni* were selected from the CHEMBL database [16,17,18] for the construction of the predictive model. The dataset consisted of 309 unique chemical structures. The molecules with pIC_50_ > 6 were considered active, and those with pIC_50_ < 6 were considered inactive, where there were 129 active and 180 inactive molecules in total.

One thousand alkaloids isolated from the families Menispermaceae and Apocynaceae were obtained from our in-house database, namely, SistematX, available at http://sistematx.ufpb.br [19,20], accessed on 28 January 2019.

For all structures, SMILES (Simplified Molecular Input Line Entry System) codes were used as input data for Marvin 18.10.0, 2018 (ChemAxon, Budapest, Hungary) [21] and Standardizer software (Chem 18.10.0, 2018; ChemAxon), Budapest, Hungary [22] to convert the chemical structures into curated and canonical representations. This standardization is of paramount importance to create consistent compound libraries and is done through the following steps: addition of hydrogens, aromatization, generation of 3D structure, and exporting the compounds in SDF format. For a more detailed description of how the dataset was curated, please refer to the workflows described by Fourches et al. [23,24,25].

### 2.2. VolSurf+ Descriptors

Molecular descriptors were generated and used to predict the biological and physicochemical properties of the molecules from the two databases after the molecules were transformed into a molecular representation. The VolSurf + v.1.0.7 software [26,27] (Molecular Discovery, Borehamwood, Hertfordshire – United Kingdom) can calculate 128 molecular descriptors using molecular interaction fields (MIFs) through N1 probes (nitrogen–hydrogen starch hydrogen bonding donor), O (acceptor hydrogen bonding), OH (water), and DRY (hydrophobic probe), as well as the calculation of non-derived MIFs.

### 2.3. Random Forest Model

The Knime 3.6.2 software (Knime 3.6.2 the Konstanz Information Miner Copyright, 2003–2019, www.knime.org, accessed on 15 December 2021, Zurich, Switzerland) [28] was used to perform the analyses and to generate the in silico model. Datasets of molecules, along with their calculated descriptors and class variables, were imported from the VolSurf+ software, program v. 1.0.7. Each dataset was divided using the “partitioning” tool with the “stratified sample” option to create a training set and an external test set, which represented 80% and 20% of the compounds, respectively. Although the compounds were selected randomly, the same proportion of active and inactive samples was maintained in both sets.

For external validation, we employed a 5-fold cross-validation using randomly selected stratified groups. The distributions according to the activity class variables were found to be maintained in all validation groups and in the training set. Descriptors were selected and modeled following a 5-fold external cross-validation procedure using the random forest algorithm (RF) [29,30]. There were 25 parameters selected for the RF for all generated models, which was the total number of trees constructed and 1 seed in the generation of random numbers for the model.

Using Knime nodes, the most important descriptors in the generation of each prediction model were evaluated. The external performances of the selected models were analyzed for sensitivity (true positive rate, i.e., active rate), specificity (true negative rate, i.e., inactive rate), and accuracy (overall predictability). The positive (PPV) and negative (NPV) predictive values informed us about the probability of predicted positives (PPV) and negatives (NPV) being the true positives and negatives, respectively. In addition, the sensitivity and specificity of the receiver operating characteristic (ROC) curve were found to describe the true performance with more clarity than accuracy.

The model was also analyzed using the Matthews coefficient, which is a way to evaluate the model globally from the results obtained from the confusion matrix. The Matthews correlation coefficient (MCC) is a correlation coefficient between observed and predictive binary classifications. It results in a value between −1 and +1, where a coefficient of +1 represents a perfect forecast, 0 is nothing more than a random forecast, and −1 indicates total disagreement between the forecast and observation [31].

The MCC can be calculated from the following formula:

(1) $$MCC=\frac{VPxVN-FPxFN}{\surd \left(VP+FP\right)\left(VP+FN\right)\left(VN+FP\right)\left(VN+FN\right)}$$

where *VP* is the number of true positives, *VN* is the number of true negatives, *FP* is the number of false positives, and *FN* is the number of false negatives.

The applicability domain (*APD*) was used to analyze the compounds of the test sets to evaluate whether their predictions were reliable. The *APD* is based on Euclidean distances, and similarity measures between the descriptors of the training set are used to define the applicability domain. This means that if a test set compound has distances and similarities beyond this limit, its prediction is not reliable. The *APD* calculation is performed using the following formula:

*APD* = *d* + *Zσ* (2)

where *d* and *σ* are the Euclidean distance and the standard mean deviation, respectively, of the compounds in the training set. *Z* is an empirical cut-off value, where, in this work, the *Z* value was set to 0.5 [32,33].

### 2.4. MuDRA Model

A second prediction model was constructed from the bank of molecules with known schistosomicidal activity to predict the activity of the alkaloid bank. The model was constructed according to the methodology of Alves et al. [34]. The model is called MuDRA and consists of instance-based machine learning, meaning that it compares new instances with seen instances in the training set rather than performing explicit generalizations. This method provides an alternative to the set-modeling approach, facilitating the implementation and lower computational cost [34,35,36].

We used the Knime software to generate the MuDRA model. We followed a 5-fold external cross-validation procedure using the complete datasets in the original publications and randomly selected 20% of the dataset as an external set using stratified sampling to standardize the analysis within this study. For the MuDRA modeling, four different types of molecular descriptors were used: Morgan, Avalon, MACCS, and RDkit. The MuDRA plating depends on the chemical similarity and comparisons of biological responses, where structural similarity is identified within a chemical space defined by the different types of molecular descriptors. In each chemical space, several nearest neighbors are selected based on their similarity to the compounds that have the known activity, and the predicted activity is calculated based on the Tanimoto coefficient as a function of the Jaccard distance [34,35].

### 2.5. Principal Component Analysis

Principal component analysis (PCA) is a chemometric tool for extracting and rationalizing the information from any multivariate description of a biological system. PCA condenses the overall information into two smaller matrices, namely, the scores plot, which shows the pattern of compounds, and the loadings plot, which shows the pattern of descriptors. PCA provides information about the relationships between samples in a data set but also gives us insight into the relationships between variables [37].

PCA studies were applied to 5 multitarget alkaloids and the 5 inactive alkaloids from the consensus analysis of the random forest and MuDRA models generated. The procedure was performed automatically by the VolSurf+ program (Molecular Discovery, Borehamwood, Hertfordshire–United Kingdom) using five principal component (PC) autoscaling and centering procedures that were applied to the PCA analysis. The utilization of PCA for dimension reduction lies in the fact that the PCs are generated so that they explain the maximal amounts of variance [27].

### 2.6. Molecular Docking

Four proteins of *S. mansoni* were downloaded from the PDB (Protein Data Bank, www.rcsb.org, accessed on 15 December 2021) [38]: *Schistosoma mansoni* 14 kDa fatty-acid-binding protein (Sm14) (transport protein, PDB ID 1VYF, ligand oleic acid) [39], histone deacetylase 8 (transferase, PDB ID 4BZ8, ligand J1038) [40], sulfotransferase (transferase, PDB ID 4MUB, ligand Oxamniquine) [41], and thioredoxin glutathione reductase (Flavoprotein, PDB ID 6FTC, ligand Hepes) [42].

To evaluate the docking procedure, we used redocking. In redocking, the position of a ligand crystallized together with the protein was used and compared with the position of the ligand docked in the active site of this same protein. The RMSD (root-mean-square deviation) was used to compare the average distance between the crystallized ligand and the ligand subjected to molecular docking, where the docking was considered valid for an RMSD of up to 2.0 Å [43]. This process was applied to the four proteins in the study.

The redocking procedure was performed using a GRID of 15 Å in radius and 0.30 in resolution to cover the ligand-binding site of both PDB files. The grid box was applied to the center of the target site and the ligand was compared with each of the other three proteins, using RMSD as an assessment metric. Templates were generated from features of both ligands that were expected to be relevant for ligand binding. The Moldock scoring algorithm was used, along with the Moldock search algorithm [44]. Molegro Virtual Docker (CLC bio Company, Aarhus, Denmark) generated five poses for each alkaloid in the active site of each protein. The most stable pose, that is, the one with the lowest interaction energy, was selected and imported to the Discovery Studio 2020 program for visual inspection [45].

The energy of the crystalized ligand of each protein is calculated automatically using the Moldock Score. In other words, from the pose of the crystallized ligand, the Moldock score algorithm automatically converts the ligand’s energy to the energy scale used by it.

After validation, the screened structures of alkaloid derivatives underwent molecular docking using the Molegro Virtual Docker software, version 6.0.1 (MVD) (CLC bio Company, Aarhus, Denmark) [46]. The alkaloid molecules were first minimized using molecular mechanics, and the most stable conformation of each alkaloid was submitted for molecular docking. All water molecules were deleted from the enzyme structures, except the thioredoxin glutathione reductase (flavoprotein, PDB ID 6FTC) in which the PDB binder has aqueous interactions. The enzyme and compound structures were prepared using the default parameter settings in the software package (ligand evaluation: Internal ES, Internal H-Bond, Sp2-Sp2 Torsions, all checked; number of runs: 10 runs; algorithm: MolDock SE; maximum interactions: 1500; max. population size: 50; max. steps: 300; neighbor distance factor: 1.00; max. number of poses returned: 5).

### 2.7. Metabolic Prediction

The three-dimensional (3D) structures of the lowest energy conformations of the 5 lead candidates were used as input data in the MetaSite 6.0 program [47] (Molecular Discovery, Borehamwood, Hertfordshire–United Kingdom). For each compound, twenty constituents were generated by the program. The metabolism sites were calculated for the liver isoforms available in the program. Then, the site of metabolism (SoM) for liver isoforms was calculated using hot-spot prediction and the structural contribution was performed in Run 32D, observing the associated MIF regions of the compounds with CYP and showing the atoms that most contribute to guiding the site of metabolism toward the heme group. Metabolites from the metabolic reaction mechanisms were identified through the metabolites identification function for liver isoforms.

### 2.8. Toxicity and Drug-Likeness Assessment

Molecular descriptors from Dragon 7.0 software [48] (Talete srl, Milano, Italy) were used to evaluate the drug-likeness properties of the five alkaloids selected in the virtual screening. To analyze the in silico toxicity of these alkaloids, the DataWarrior v4.7.2 software [49] called OpenMolecules (http://www.openmolecules.org/datawarrior/download.html, accessed on 15 December 2021) was used, which evaluates the mutagenic, tumorigenic, reproductive, and irritant effects.

## 3. Results and Discussion

### 3.1. Ligand-Based Virtual Screening

The bank of molecules with known activity against *S. mansoni* and the alkaloids isolated from the Menispermaceae and Apocynaceae families were described using five different molecular descriptor types and predictions were made using models built following the best practices of QSAR (quantitative structure–activity relationship) modeling [30,50].

The RF model was generated following the fivefold cross-validation procedure [29,30], which means that the entire data set was partitioned five times into a modeling set (training set), including 80% of the compounds the set, and the external cross-validation data set comprised the remaining 20% of the compounds the data set. After this, only the modeling set was used to build the models, and then the models are validated with the external cross-validation technique. In Table 1, it is possible to observe the confusion matrices for each model in the external cross-validation and the statistical performance variability between the models.

**Figure 1:**
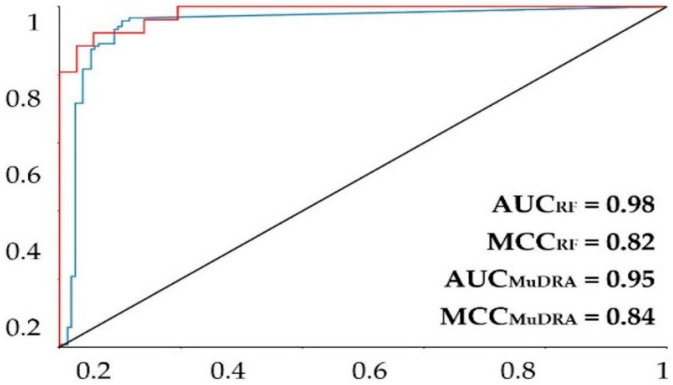
ROC plot for the MuDRA and RF models. AUC—value of the area under the curve; MCC—Matthews correlation coefficient. Red line—RF 5-fold external test cross-validation; blue line—MuDRA fivefold external test cross-validation.

For the model built using the random forest (RF) algorithm, the model obtained a good prediction rate (Table 2), with an accuracy of more than 0.9, revealing a robust model. The performance of the model was further evaluated with the ROC curve and the MCC. The area under the curve obtained for the model was 0.92 for the fivefold external test set (Figure 1). The MCC was 0.77 for the fivefold external test set. Knowing that a perfect model has an area under the curve equal to 1, it is possible to state that the models above can perform a good classification rate for this RF method.

Of the 1000 alkaloids analyzed in the model, 993 were within the chemical space, i.e., within the applicability domain of the generated prediction model, and therefore, their predictions were reliable. Of the 993 alkaloids that were within the applicability domain, only 32 were predicted as active, with their predictions of activity varying between 50 and 73% probability.

The MuDRA model was generated using four different types of molecular descriptors—RdKit, Avalon, Morgan, and MACS KEYS—and obtained similar metrics to the RF model. From Table 2, it is clear that the model had a good prediction rate, with hit rates higher than 91% for the external cross-validation, revealing a good model. The model showed an area under the curve of 0.95 and an MCC of 0.85 (Figure 1), which are extremely high statistics, indicating a robust model. The MuDRA model indicated 277 alkaloids with probabilities between 52 and 65% for being active against *S. mansoni*. These molecules had similarities between 0.89 and 0.45 with chemical structures that have known schistosomicidal activity. Of the 277 alkaloids selected from the MuDRA model, 20 had a probability of activity higher than 60% and similarities between 0.51 and 0.86.

To select the alkaloids that were most likely to be active against *S. mansoni* from the two predictive models generated in this study, a consensus analysis of the ligand-based virtual screening was performed using the following formula:

(3) $$P_{cm}=\frac{\left(p_{RF}*ESP_{RF}\right)+\left(p_{MuDRA}*ESP_{MuDRA}\right)}{\left(ESP_{RF}+ESP_{MuDRA}\right)}$$

where *P_cm_* is the combined probability between the models, *P_RF_* is the probability of the alkaloid being active in the random forest model, *ESP_RF_* is the specificity of the *RF* model, *P_MuDRA_* is the probability of the MuDRA model, and lastly, *ESP_MuDRA_* is the specificity of the MuDRA model. In this equation, the score of the activity probabilities of each model is conditioned by a decrease in the rate of false positives with the increase in specificity. Thus, the probability of selecting inactive molecules as active molecules is minimized [12,51,52,53].

From this consensus analysis, we selected 61 alkaloids that were likely to be active with a probability greater than 60% from the two predictive models. Through this analysis, we increased the probability of selecting more potentially active molecules since they have a high probability of being active in two different virtual screening approaches.

### 3.2. Structure-Based Virtual Screening

With the alkaloids identified from our QSAR modeling, we started the structure-based virtual screening (using molecular docking) to simulate their interactions in the active site of the four *S. mansoni* target proteins, which were chosen to analyze four different mechanisms of action with a schistosomicidal response.

Table 3 provides information on the lowest interaction energies of the alkaloid obtained from docking (best E_ALK_), the RMSD redocking, and the interaction energies (MoldockScore) of the PDB binder of each protein (E_ligPDB_). In this redocking analysis, it was observed that all RMSD values were below 2.0 Å (Table 2), showing a good prediction of the redocking simulation (Appendix A—Figure A1) [54].

After the validation, the molecular docking was performed for 61 alkaloid compounds to simulate their interactions with four enzymes of *S. mansoni*: *Schistosoma mansoni* 14 kDa fatty-acid-binding protein (Sm14) (PDB ID 1VYF), histone deacetylase HDAC8 (PDB ID 4BZ8), sulfotransferase (PDB ID 4MUB), and thioredoxin glutathione reductase (PDB ID 6FTC) (Appendix A—Table A1).

Some of the alkaloids demonstrated lower docking energies when compared with the ligand PDB, suggesting that these alkaloids could have a stronger interaction with the active site amino acids residues of the respective enzymes.

For the *Schistosoma mansoni* 14 kDa fatty-acid-binding protein (Sm14) (PDB ID 1VYF), the alkaloid dehatridine exhibited the lowest values of docking (−147.1 kcal/mol), with a more favorable docking score than the PDB ligand oxamniquine (−86.6 kcal/mol). For oxamniquine, we found that Tyr131, Arg129, and Tyr118 were the key residues involved in its activity. Similarly, there were 12 key interactions between the alkaloid and the enzyme: six were H-bond interactions (Ile32, Phe57, Ser55, Ser75, Tyr129, and Gln94) and six were pi interactions (Pro38, Leu60, Arg127, Phe16, Met20, and Val25). This similarity between oxamniquine and dehatridine could indicate a similar action and activity (Appendix A—Figure A2).

Likewise, the alkaloid dauricoside demonstrated a strong interaction with the histone deacetylase 8 (PDB ID 4BZ8). This binding energy value was −167.7 kcal/mol, which was more favorable than that of the PDB ligand (−81.84 kcal/mol). This can be explained by a higher number of H-bond interactions in the presence of methoxy and hydroxyl groups: Asp285, His142, Asp100, and His292. Furthermore, the pi interactions were present in aromatic and cyclic forms, with His188 and Phe216 key residues (Appendix A—Figure A2).

For sulfotransferase (PDB ID 4MUB), secohomoaromoline had the lowest energy value (−154.8 kcal/mol), which again demonstrated more favorable binding energy than the PDB ligand (−71.5 kcal/mol). The PDB ligand’s key interacting residues included H-bonds to Pro225, Cys226, Lys21, Trp18, Gly19, Asn228, and Val227; pi-sigma bonds to Leu203; and salt bridges to Arg15 and Arg17 in aromatic and PO^4−^ fragments. The alkaloids demonstrated a more favorable binding energy score, which was likely due to the presence of pi interactions in aromatic, methoxy, and hydroxyl fragments, providing a total of 16 interactions with the compounds. However, these similar interactions could indicate a similar interaction activity with the protein (Appendix A—Figure A2).

For the flavoprotein thioredoxin glutathione reductase (PDB ID 6FTC), the alkaloid stesakine 9-o-b-d-glucoside presented more favorable binding interactions (−125.8 kcal/mol) than the PDB ligand (−72.7 kcal/mol). For this protein, we identified many hydrogen bonds between the water molecules and amino acid residues and observed conserved interactions between the residues Tyr479, Asp325, and Gly323 between the alkaloid and the PDB ligand, which may indicate a similar interaction activity with the protein (Appendix A—Figure A2).

### 3.3. Consensus Analysis

For the four proteins used in this study, the favorable binding affinities of the alkaloids could be explained by the greater presence of steric and H-bond interactions with the ketone, methyl formate, and hydroxyl groups, conferring strong interactions with the study enzymes. Based on the binding energy values, all tested molecules were ranked using the following probability calculation:

(4) $$Ps=\frac{E_{TM}}{E_{M}},IFE_{TM}<E_{LigPDB}$$

where *p_s_* is the structure-based probability, *E_TM_* is the docking energy of molecule test and *TM* ranges from 1 to 61 (the alkaloids selected from consensus analyzes the ligand-based), *E_M_* is the molecule that has the lowest value of the energy in the MoldockScore of the dataset, and *E_LigPDB_* is the ligand energy from protein crystallography [12,51,52,53].

This equation aims to normalize the scores obtained from molecular docking (structure-based virtual screening) so that the values can be compared with the active probability values from the ligand-based virtual screening. In addition, a principle of selection is that the structures must have an energy lower than the value obtained for the ligand in the crystallography study. The alkaloids were classified as active if the structure-based probability values were greater than or equal to 0.65.

The numbers of molecules with probability values greater than 0.65 and binding energy values less than the ligand were 1VYF (29), 4BZ8 (12), 4MUB (30), and 6FTC (33).

We used an approach of combining structure-based and ligand-based virtual screening to verify potentially active molecules, as well as their possible mechanism of action, showing potential multitarget molecules. This approach also sought to minimize the likelihood of selecting false-positive molecules, as it also considered the specificity rate of ligand-based virtual screening techniques. The calculations were done with the following equation:

(5) $$Pc=\frac{\left(P_{s}+\left(1+ESP_{med}\right)\times P_{cm}\right)}{\left(2+ESP_{med}\right)}$$

where *P_c_* is the approach combining probability, *P_s_* is the structure-based probability, *ESP_med_* is the media specificity rate between the two models, and *P_cm_* is the probability combined between the models [12,51,52,53].

**Figure 2:**
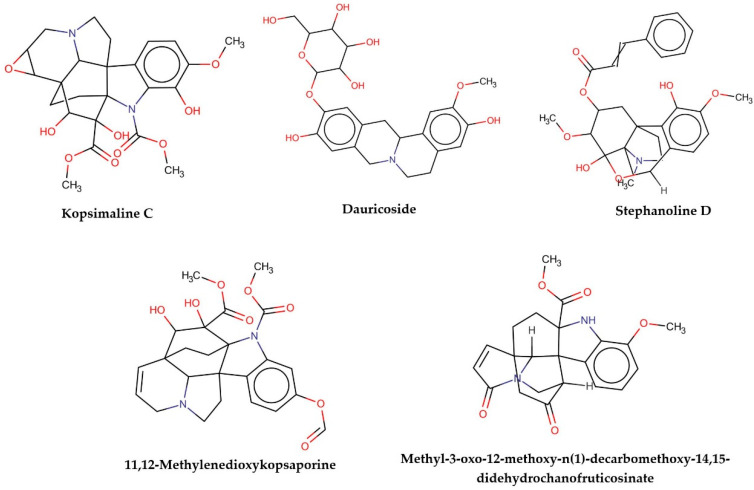
Consensus hits from the structure-based and ligand-based virtual screening.

**Figure A4:**
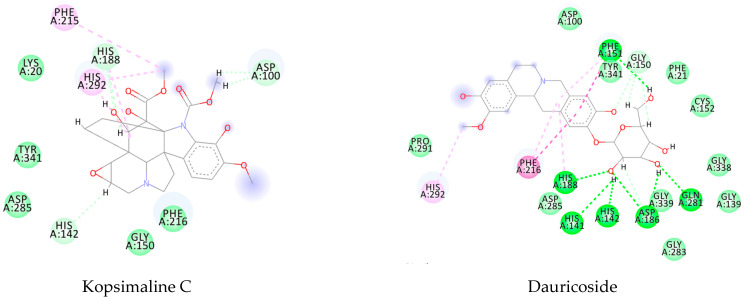

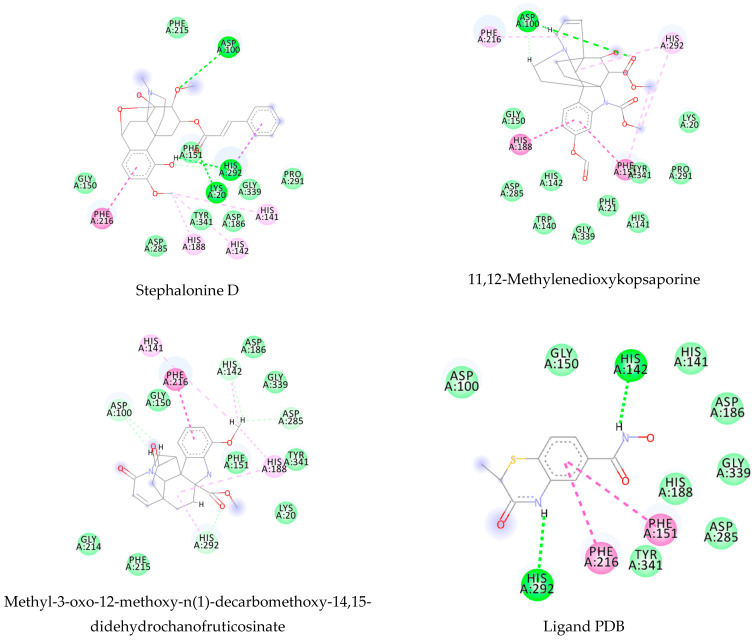
Molecular docking interactions between the five selected VS hits and the 4BZ8 protein. The light green represents the van der Waals interactions, the green represents the conventional H-bonds, and the pink represents the pi-interactions.

**Figure A5:**
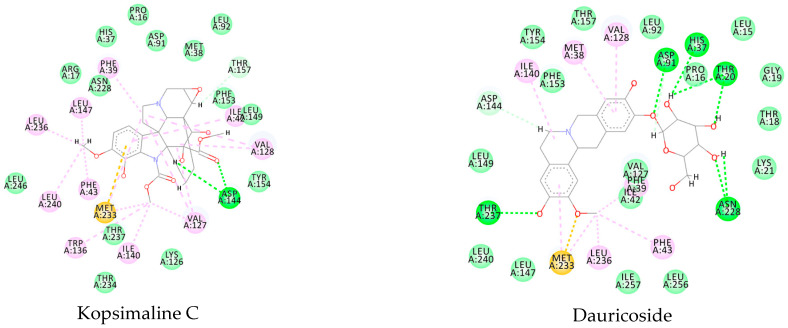

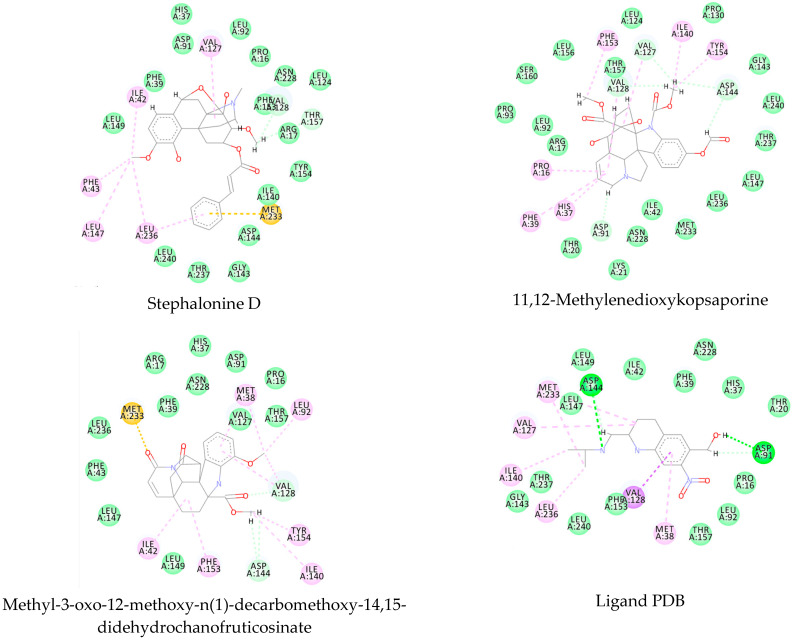
Molecular docking interactions between the five selected VS hits and the 4MUB protein. The light green represents the van der Waals interactions, the green represents the conventional H-bonds, the pink represents the pi-interactions, and the orange represents the pi-sulfur interactions.

**Figure A6:**
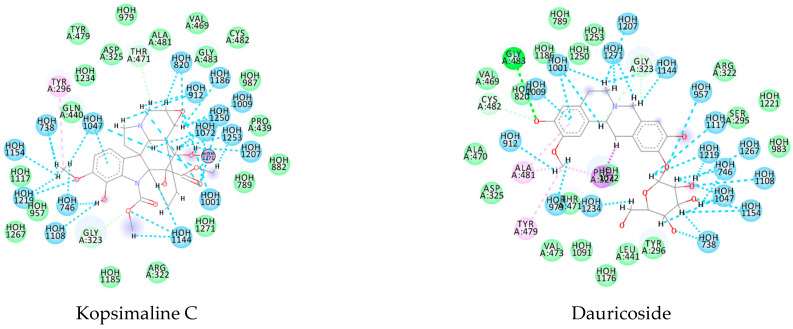

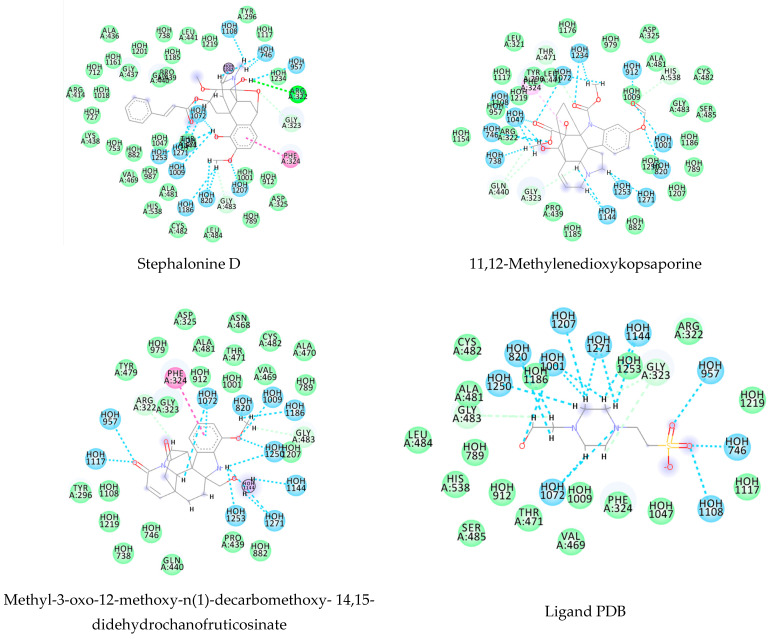
Molecular docking interactions between the five selected VS hits and the 6FTC protein. The light green represents the van der Waals interactions, the green represents the conventional H-bonds, the pink represents the pi-alkyl interactions, the purple represents the pi-sigma interactions, and the blue represents the water H-bonds.

This consensus analysis was performed for each protein studied. Then, the five alkaloids with the highest probability of interacting with the four proteins were selected, therefore making them multitarget and with a greater potential of schistosomicidal activity. Table 4 summarizes the results for these five alkaloids obtained using the combined approach, and Figure 2 shows the chemical structures of these alkaloids. These alkaloids interacted in the active site of all proteins analyzed, with them being potential multitarget alkaloids, and mostly had carbon–hydrogen interactions and H-bonds with the amino acid residues at the active site of the proteins (Appendix A—Figure A3, Figure A4, Figure A5 and Figure A6).

It was observed that four compounds presented an indolic structural fragment, with dauricoside being the exception. Further, the presence of hydroxyl, methoxy, and ester groups was favorable for the activity, which was verified as a fundamental part in promoting interactions with *S. mansoni* proteins.

The two QSAR models generated in this study were robust and predictive, with accuracies for the sets ranging from 87 to 91%. It can also be observed that the models obtained high rates of sensitivity and specificity, revealing that they learned to distinguish the classes well, obtaining low rates of false positives and false negatives and consequently high rates of PPV and NPV.

For a better analysis of the biological activity structure of these compounds selected in the combined analysis of ligand- and structure-based virtual screening, a PCA study was performed that compared the five alkaloids selected with alkaloids that were less likely to be inactive.

The PCA results were based on the interactions of 3D structures and a GRID force field using all four probes—H2O, DRY, N1, and O—where 128 molecular descriptors were calculated. The data was autoscaled (preprocessed) and the PCA was performed on the dataset of 10 alkaloids with potential antischistosomal activity: five of the alkaloids had multitarget activity and the other five alkaloids had inactivity against *S. mansoni*. The PCs were constructed in a way that the first few components described most of the variance among descriptors. Therefore, in our studies, we observed that PC1 and PC2 explained 65% of total variance from the original data using 128 descriptors.

**Figure 3:**
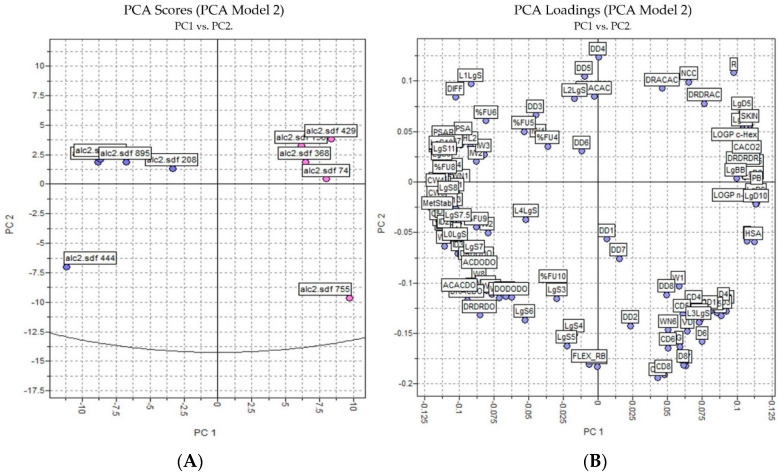
PCA analysis applied to the more active and less inactive alkaloids: (**A**) score plot (blue—active alkaloids, red—inactive alkaloids) and (**B**) loading plot.

Analyzing Figure 3, we observed that the inactive and active alkaloids were more distinct for PC1 and not for PC2, with the coefficients of PC1 being the most informative. Moreover, the PCA score showed good separation between the more active alkaloids (in blue) and the inactive alkaloids (in red), demonstrating that the structural difference between the compounds may correspond to their biological activity.

In the loading plot, we observed several descriptors with high relevance to the structure of the most active compounds and were considered positive characteristics for the activity of these compounds: percentage of unionized species (FU%), 3D pharmacophoric descriptors (DODODO, ACACDO, DRDRDO), solubilities at various pH (LgS), metabolic stability with human CYP3A4 enzyme (MetStab), hydrophilic volume over the area (PSA), and center of mass to the hydrophilic regions (IW).

Some of these descriptors were also observed in the RF model (available in PMML file upon request) as high relevance. Descriptors like IW, LgS, MetStab, and ACACDO obtained Cohen’s kappa coefficients of 0.818, 0.727, 0.636, and 0.545, respectively. Cohen’s kappa coefficient (κ) is a statistic that is used to measure inter-rater reliability (and intra-rater reliability) for qualitative (categorical) items [55]. It is generally thought to be a more robust measure than a simple percent agreement calculation, as κ takes into account the possibility of the agreement occurring by chance [55].

**Figure 4:**
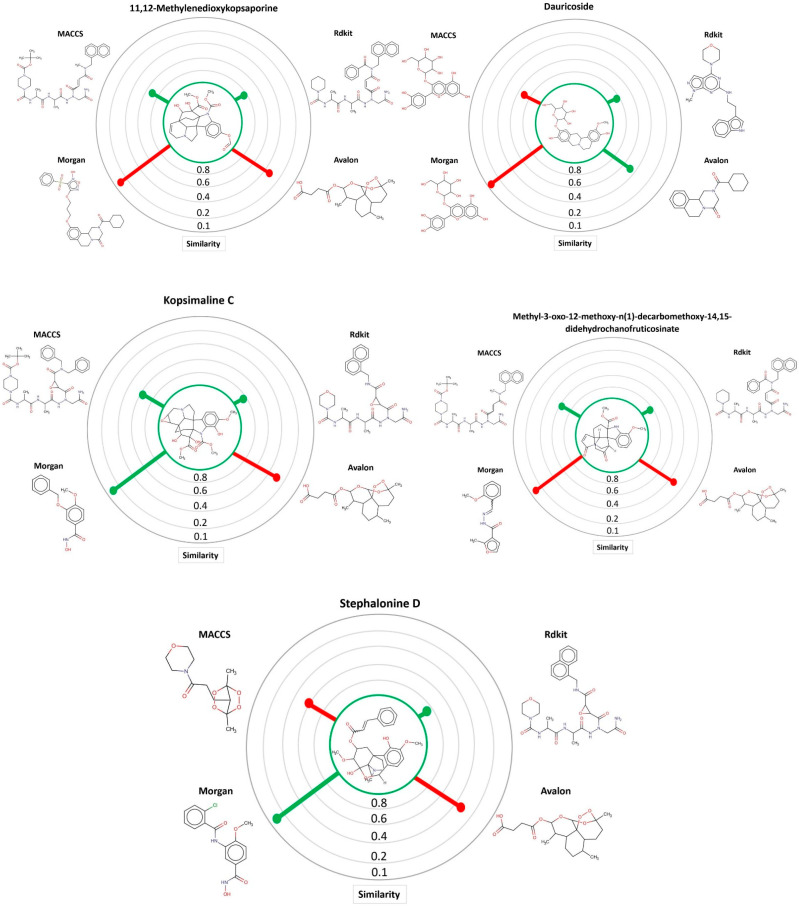
MuDRA plots for five consensus hits. Green—similarity to active compounds against *S. mansoni* and red—similarity to inactive compounds against *S. mansoni*.

Through the MuDRA model, it was possible to analyze the similarity for the five multitarget alkaloids with the bank of molecules of known activity against *S. mansoni*. In Figure 4, one can see the five alkaloids and their similarities with the active and inactive compounds according to four different types of molecular descriptors.

**Figure 5:**
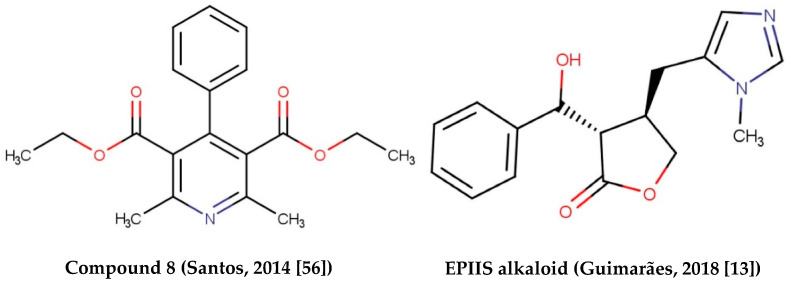
Alkaloids with schistosomicidal activity found in the literature.

Alkaloids with schistosomicidal activity similar to those in our study were reported in the literature. Santos et al. [56] reported the identification of alkaloid diethyl 4-phenyl-2,6-dimethyl-3,5-pyridinedicarboxylate (Figure 4), isolated from *Jatropha elliptica*, with 100% lethal activity (50 μg/mL) against adult *S. mansoni*. Guimarães et al. [13] reported the action of Epiisopilosine alkaloid (Figure 5), which at a 100 mg/kg dose administered orally against *S. mansoni* in mice, was effective up to 60.61%. In addition, the authors reported no in vitro cytotoxicity (in HaCaT and NIH-3T3 cells) up to 512 μg/mL, and showed that EPIIS had low predicted toxicity in silico using pkCSM software. Rocha et al. [57] performed an in silico method using ADMET predictions and molecular docking applied to five imidazole alkaloids derivates: EPI (epiisopiloturine), EPIIS (epiisopilosine), ISOP (isopilosine), PILO (pilosine), and MAC (Macaubine). The compounds showed high stability, and with the EPIIS present, the best values from the molecular docking experiments corroborated with the results of experimental studies on schistosomiasis.

### 3.4. Prediction of Metabolism of Selected Alkaloids

**Figure 6:**
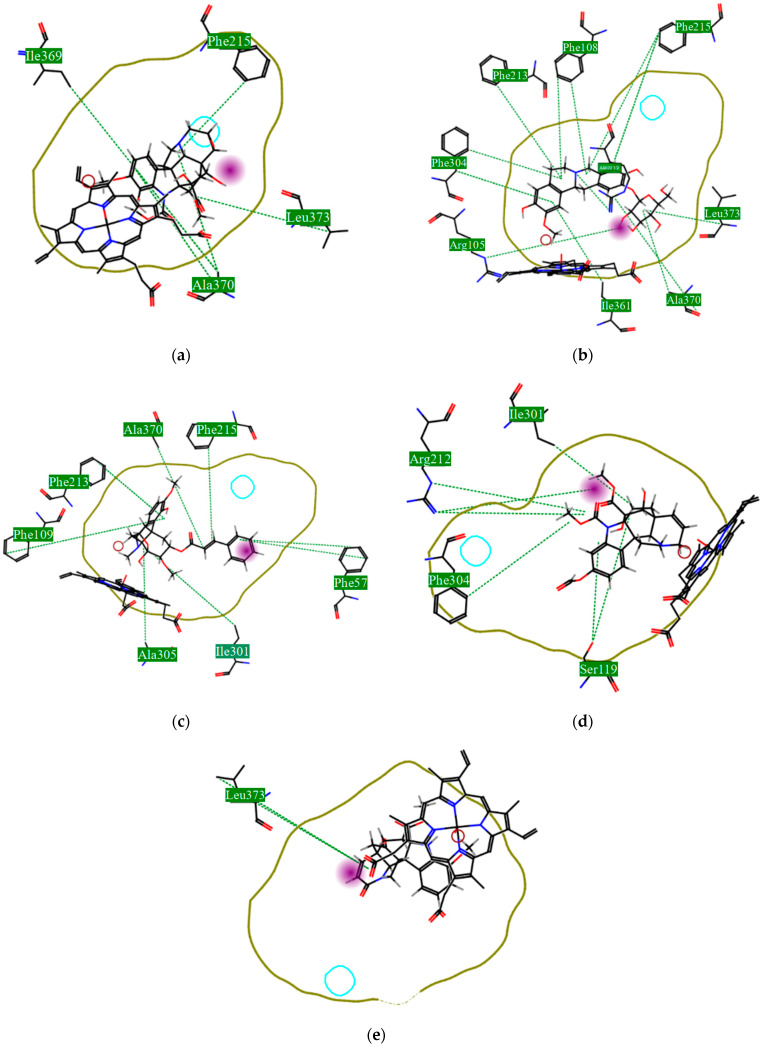
Structural contribution of the molecular coupling between the leading compounds and the liver isoform 3A4 in (**a**) kopsimaline C, (**b**) dauricoside, (**c**) stephalonine D, (**d**) 11,12-methylemedioxykopsaporine and (**e**) methyl-3-oxo-12-methoxy-n(1)-decarbomethoxy-14,15-didehydrochanofruticosinate.

Using MetaSite v. 6.0, we evaluated the potential site of hepatic metabolism using cytochrome P450 regulated biotransformations in silico. According to the analysis, it was possible to observe the highest probability of metabolization at the carbonic portions close to oxygen, such as methoxylates and hydroxyls (Figure 6). It was observed that a large number of amino acids present at the active site of the cytochrome had hydrophobic characteristics, demonstrating the characteristic of similarity between the five compounds studied here. Except for the hydrophobic amino acids, only arginine (basic polar) and serine (neutral polar) were present in the compounds dauricodise and 11,12-methylethylmethoxykopsaporine.

We also observed that the position at the cytochrome active site was the same for the compounds kopsimaline C, dauricoside, and stephalonine D (Figure 6), with common interactions at the amino acid residues Leu373, Phe215, and Ala370. These may be residues that are critical for the interaction and stabilization of the anchoring pose with the cytochrome. In fact, the interactions with these residues were present in regions close to oxygen-containing groups (such as ketones, hydroxyls, and carboxylic acids), as well as nitro and aromatic rings in their structure. On the other hand, the compounds 11,12-methylemediatexykopsaporine and methyl-3-oxo-12-methoxy-n(1)-decarbomethoxy-14,15-didehydrochanofruticosinate showed molecular coupling divergences, presenting two interactions in common, namely, Leu373 and Arg212, demonstrating their structural template differences in the cytochrome. Therefore, interactions with the amino acid residues demonstrated a coupling difference between the compounds of the study, which could result in significant differences in the biotransformation process.

This characteristic could be correlated with observations of metabolites from the biotransformation with cytochrome 3A4 present in the liver. It was observed that there was a reactional difference between the five lead compounds regarding the types of reactions that could be linked to the active site and interaction with the cytochrome heme group. From this, it was possible to observe the lower occurrence of iminium formation reactions, aliphatic carbonylation, alcoholic oxidation, aliphatic hydroxylation, and aromatic hydroxylation (Table 5).

On the other hand, the higher occurrence of dealkylation reactions may indicate the prevalence of this type of reaction in the study compounds. This reaction is carried out via the removal of alkyl groups bound to nitrogen ring atoms and removed via oxidation in the formation of aldehydes.

The presence of the heme group in the cytochrome framework functions as an electron transfer chain, converting the structure of the drug into a more water-soluble substance. In our analysis, it was possible to note that the compounds dauricoside, stephalonine D, and 11,12-methylemedioxykopsaporine did not interact with the heme group present in the enzyme, while the compounds kopsimaline C and methyl-3-oxo-12-methoxy-n(1)-decarbomethoxy-14,15-didehydrochanofruticosinate showed interactions with the heme group, making the metabolism of these two latter compounds more rapid. According to Devlin et al. [58], a complex formed between a compound and the heme group can result in a conformational change in the enzyme, allowing for molecular oxygen fixation and the transfer of electrons, finalizing the function of facilitating the excretion of xenobiotics. Therefore, according to the reactions, it was possible to observe that this process may provide the activation of compounds 4 and 5 and that its possible metabolization occurs in a smaller way in the organism.

It was also possible to observe that the metabolic modifications caused in the structure of the five lead compounds did not interfere with the large structural influence portion, which was demonstrated as a factor of great essentiality for the biological activity. In this way, it is possible to observe that metabolites can also present biological activity.

### 3.5. Drug-like and Toxicity Analyzes

To analyze whether the five alkaloids selected had a good profile to be drug-like, predictions of their toxicity in the human body were calculated using some molecular descriptors and analyzed using the Lipinski rules.

First, the logP of the compounds was analyzed, where logP measures the hydrophilicity profile of a molecule. Drugs intended for oral use should have a logP within the range of 1 to 3, which will have optimal pharmacokinetic and pharmacodynamic requirements for oral administration [27,32]. The selected alkaloids—kopsimaline C, dauricoside, stephalonine D, 11,12-methylethylenedioxypropoxy, and methyl-3-oxo-12-methoxy-n(1)-decarbomethoxy-14,15-didehydrochanofruticosinate—obtained the following logP values: 0.04, −0.26, 2.13, 1.14, and 1.72, respectively.

After calculating the logP values, other parameters from the Lipinski alert index were evaluated in silico using the Dragon 7.0 software. This alert is the first filter to predict the oral bioavailability of compounds that achieve the clinical status of phase II and predict that malabsorption or permeation is more likely when more than one of the rules is violated [59,60,61,62,63]. In this analysis, no compound was removed, as there was no violation of any rule.

Then, the consensus drug-likeness scores were analyzed, where this uses an average of seven drug-likeness indexes already on the market [61,62,63,64,65,66], that is, they are parameters that have model variables used as filters to select candidates for drugs. From this analysis, the alkaloids stephalonine D, 11,12-methylethylmethyl-xykosaporine, and methyl-3-oxo-12-methoxy-n(1)-decarbomethoxy-14,15-didehydrochanofruticosinate were prominent as promising drug candidates, with 11,12-methylethylmethyl-xykosaporine being the most promising among them.

In addition, the potential toxicity of these molecules was calculated using DataWarrior v4.7.2, OpenMolecules (http://www.openmolecules.org/datawarrior/download.html, accessed on 5 December 2021).

DataWarrior is a program that performs in silico chemical analysis through a database. It is possible, among other analyses, to generate predictions of the toxicity of chemical compounds [49]. This prediction is performed using algorithms and a bank of fragments with known toxicities [49]. Thus, when we insert the molecules that we want to calculate an in silico toxicity profile for, the DataWarrior software will make this comparison of chemical fragments between our molecules and fragments of known toxicities, thus generating a prediction about the toxicity profile of the molecules in analyses. The mutagenicity, tumorigenic, reproductive toxicity, and dermal toxicity were analyzed. The predictions are classified as low risk, medium risk, and high risk. The results indicated that the alkaloid stephalonine D presented a high risk of skin irritation, while the others did not show any toxicity. The results of these analyses can be observed in Table 6.

## 4. Conclusions

Using an integrated computational approach for the virtual screening of 1000 alkaloids obtained from the SistematX database, we identified five alkaloids from the Apocynaceae and Meninspermaceae families with potential multitarget schistosomicidal activity. Two of them, namely, 11,12-methylethylenedioxypropoxy and methyl-3-oxo-12-methoxy-n(1)-decarbomethoxy-14,15-didehydrochanofruticosinate, also had plausible toxicity and metabolites profiles. These proposed hits could serve as a promising starting point for the development of new schistosomicidal compounds based on natural products.

## Figures and Tables

**Table 1:** Statistical performances and confusion matrices for the RF model.

	**Modeling Set**		**External Cross-Validation**			
**Fold**	**nº Compounds**	**Accuracy (%)**	**No. Compounds**	**Accuracy (%)**	**Sensitivity (%)**	**Specificity (%)**
**1**	247	89	62	90	96	86
**2**	247	90	62	89	89	89
**3**	247	89	62	90	92	89
**4**	247	91	62	90	88	91
**5**	248	92	61	91	85	97
	**Confusion Matrices—External Cross-Validation**					
	**Fold**	**No. Compounds**	**True Positive**	**False Positive**	**True Negative**	**False Negative**
	1	62	25	5	31	1
	2	62	23	4	32	3
	3	62	24	4	32	2
	4	62	23	3	33	3
	5	61	22	1	35	3

**Table 2:** Summary of parameters corresponding to the results obtained for all models.

Models	Specificity	Sensitivity	Accuracy	PPV	NPV
RF	0.91	0.90	0.90	0.88	0.92
Mudra	0.90	0.93	0.91	0.88	0.94

**Table 3:** Summary of parameters corresponding to the results obtained for all models.

Protein Name	PDB ID	Best EALK	RMSD	EligPDB (Crystallized Ligand)	EligPDB (Redocking)
*Schistosoma mansoni* 14 kDa fatty-acid-binding protein (Sm14)	1VYF	−147.1 ^1^	0.51	−88.53	−86.63
Histone deacetylase 8	4BZ8	−167.70 ^2^	0.22	−85.97	−81.84
Sulfotransferase	4MUB	−154.80 ^3^	0.26	−74.81	−71.55
Thioredoxin glutathione reductase	6FTC	−125.82 ^4^	0.48	−76.24	−72.78

^1^ Dehatridine; ^2^ dauricoside; ^3^ secohomoaromoline; ^4^ stesakine-9-o-b-d-glucoside.

**Table 4:** Consensus hits of the five alkaloids from structure- and ligand-based virtual screening; *P_cm_* is the combined probability between the models in ligand-based VS, *Ps* is the probability value in the structure-based VS, and *P_c_* is the combined approach probability.

Molecule	*P_cm_*	Index	1VYF	4BZ8	4MUB	6FTC
Kopsimaline C	0.73	*P_s_* *P_c_*	0.80 0.76	0.64 0.70	0.83 0.77	0.92 0.80
Dauricoside	0.72	*P_s_* *P_c_*	0.82 0.75	1 0.82	0.84 0.76	0.77 0.74
Stephalonine D	0.70	*P_s_* *P_c_*	0.81 0.74	0.87 0.76	0.84 0.75	0.89 0.77
11,12-Methylenedioxykopsaporine	0.69	*P_s_* *P_c_*	0.66 0.68	0.83 0.74	0.68 0.69	0.87 0.75
Methyl-3-oxo-12-methoxy-n(1)-decarbomethoxy-14,15-didehydrochanofruticosinate	0.64	*P_s_* *P_c_*	0.74 0.68	0.66 0.65	0.71 0.67	0.70 0.66

**Table 5:** Secondary metabolites and types of reactions from the molecular docking with the cytochrome of the liver: (a) kopsimaline C, (b) dauricoside, (c) stephalonine D, (d) 11,12-methylemedioxykopsaporine, and (e) methyl-3-oxo-12-methoxy-n(1)-decarbomethoxy-14,15-didehydrochanofruticosinate.

Substrate	Metabolite 1	Metabolite 2	Metabolite 3	Metabolite 4	Metabolite 5
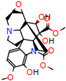 **a**	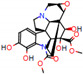 O-Dealkylation	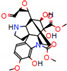 N-Dealkylation	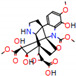 N-Dealkylation	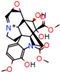 Iminium Formation	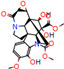 Aliphatic Carbonylation
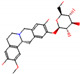 **b**	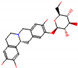 O-Dealkylation	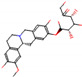 O-Dealkylation	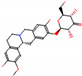 Alcoholic Oxidation	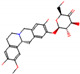 Alcoholic Oxidation	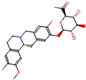 Alcoholic Oxidation
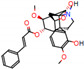 **c**	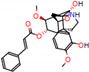 N-Dealkylation	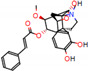 O-Dealkylation	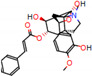 O-Dealkylation	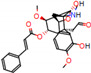 N-Dealkylation	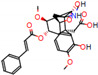 N-Dealkylation
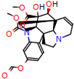 **d**	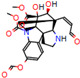 N-Dealkylation	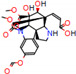 N-Dealkylation	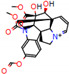 Iminium Formation	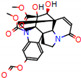 Aliphatic Carbonilation	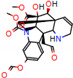 N-Dealkylation
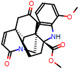 **e**	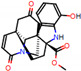 O-Dealkylation	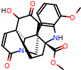 Aliphatic Hydroxylation	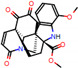 Aliphatic Hydroxylation	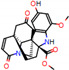 Aromatic Hydroxylation	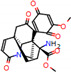 N-Dealkylation

**Table 6:** Toxicity analyses of the selected alkaloids.

Molecule	Mutagenic	Tumorigenic	Reproductive Toxicity	Dermal Toxicity
Kopsimaline C	None	None	None	None
Dauricoside	None	None	None	None
Stephalonine D	None	None	None	High risk
11,12-Methylenedioxykopsaporine	None	None	None	None
Methyl-3-oxo-12-methoxy-n(1)-decarbomethoxy-14,15-didehydrochanofruticosinate	None	None	None	None

**Figure A1:**
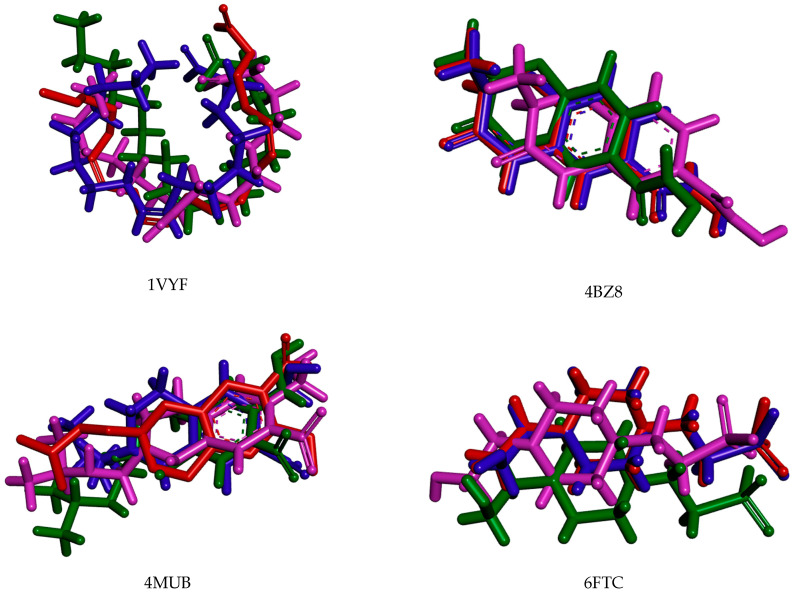
Redocked structures of PDB ligands in the active site of each studied protein. The red represents the cocrystal ligand, the blue represents the best pose for redocking, the pink represents the second-best pose for redocking, and the green represents the third-best pose for redocking.

**Figure A2:**
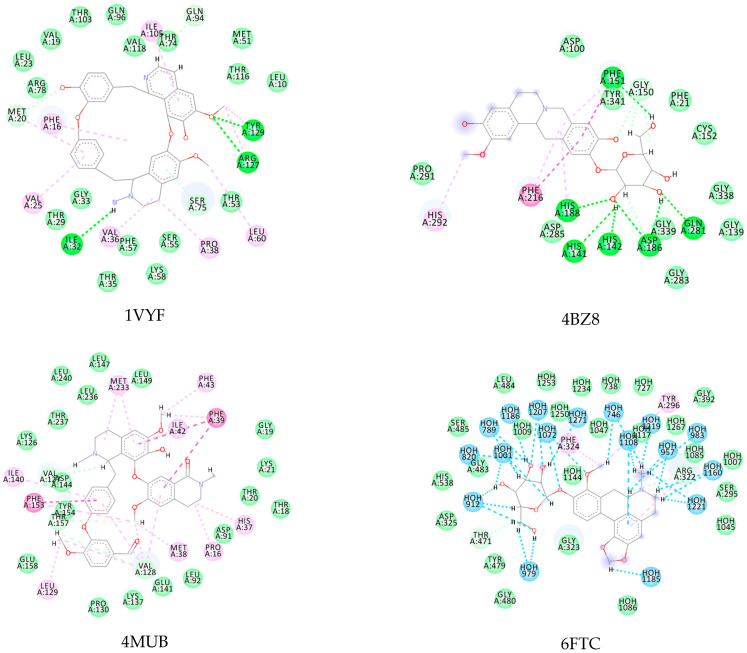
Alkaloids with the lowest energy values regarding molecular docking. The light green represents the van der Waals interactions, the green represents the conventional H-bonds, the pink represents the pi interactions, and the blue represents the water H-bond interactions.

**Figure A3:**
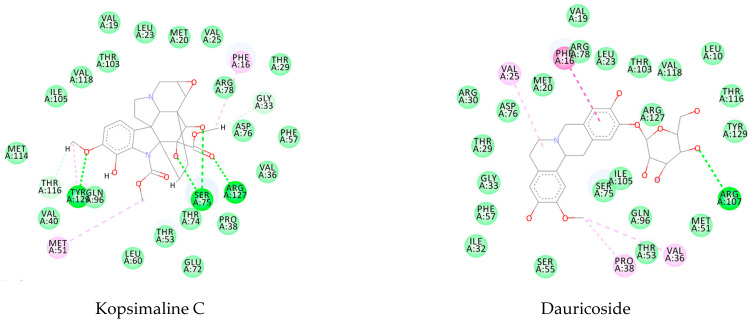

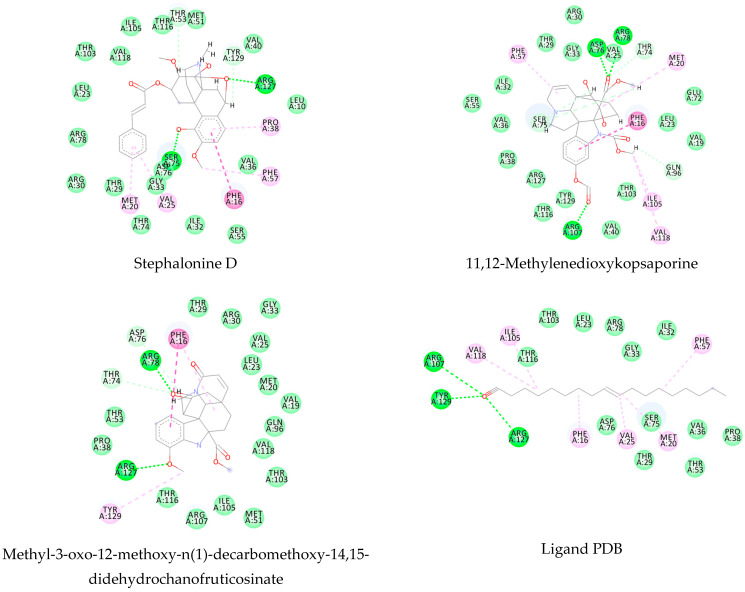
Molecular docking interactions between the five selected VS hits and the 1VYF protein. The light green represents the van der Waals interactions, the green represents the conventional H-bonds, and the pink represents the pi-interactions.

## Data Availability

Not applicable.

## Appendix A

**Table A1:** Molecular docking results of 61 alkaloids in four *S. mansoni* proteins. Energy values were quantified in kilocalories per mole.

Compound_Name	1VYF	4BZ8	4MUB	6FTC
Bisaknadinine	−11.72	−82.27	−66.68	−63.46
Nitaphylline	−93.64	−119.96	111.63	4.72
Leucolusine	−91.23	−45.91	−75	−81.92
Stesakine 9-O-b-D-glucoside	−98.81	−128.85	−136.44	−125.82
Bisinomenine	−109.73	29.43	−90.57	20.6
Kopsimaline C	−119.07	−108.56	−129.61	−88.98
Dauricoside	−121.12	−167.78	−130.14	−111.4
Kopsimaline F	−47.47	−99.61	−118.64	−88.49
Mersilongine	−51.53	−92.85	−97.84	−41.96
Mersilfoline B	−55.62	−70.08	−78.49	−37.56
Stephalonine D	−120.18	−147.16	−130.34	−88.71
Kopsifoline C	−92.87	−82.48	−131.52	−63.74
Kopsiloscine D	−108.95	−96.57	−110.53	−96.6
Kopsingine	−91.06	−90.3	−100.04	−44.05
11,12-Methylenedioxykopsaporine	−97.19	−140.91	−106.58	−89.29
Voachalotine oxindole	−71.4	−75.38	−104.95	−66.48
11,12-Methylenedioxykopsinol	−100.82	−110.79	−128.9	−74.89
Kopsidasine n-oxide	−49.93	−54.62	−64.24	−15.24
Stephalonine E	−95.68	−108.73	−96.83	−71.08
Kopsifoline b	−89.52	−75.85	−121.72	−60.65
Affinine	−99.97	−98.56	−109.07	−106.94
Jerantinine c	−96.02	−108.01	−112.42	−81.91
12-Demethoxykopsingine	−93.37	−110.82	−93.47	−50.82
Kopsimaline a	−123.58	−139.07	−89.9	−112.8
Valesamina	−96.14	−94.62	−90.52	−96.13
Jerantinine b	−91.65	−104.49	−112.84	−96.27
Prunifoline f	−103.05	−87.92	−92.2	−21.34
Kopsiloscine e	−117.29	−79.36	−118.67	−65.31
12-Methoxykopsinaline	−92.33	−76.26	−95.59	−29.34
Jerantinine d	−99.78	−108.72	−120.78	−71.75
Methyl-3-oxo-12-methoxy-n(1)-decarbomethoxy- 14,15-didehydrochanofruticosinate	−109.89	−112.23	−110.54	−89.49
Secohomoaromoline	−116.04	−129.7	−154.8	−97.94
Kopsidarine	−73.72	−96.13	−101.26	−46.91
Alpneumine h	−61.29	−93	−84.58	−103.19
Aknadilactam	−88.94	−101.18	−83.19	−38.56
Lapidilectinol	−85.2	−89.25	−72.05	−47.22
Mersidasine c	−88.12	−60.28	−84.52	−33.61
Telikovinone	−62.13	−64.49	−91.73	−39.23
Kopsiloscine B	−100.81	−105.86	−87.98	−87.67
Disinomenine	−139.05	−110.51	−92.5	−25.65
Lahadinine B	−95.1	−88.11	−75.79	−36.6
Neotrilobine	−104.96	−94.71	−88.33	−103.53
Kopsofinone	−113.3	−76.16	−101.8	−62.85
N-oxide	−89.86	−78.11	−76.28	−40.13
Kopsiloscine j	−93.87	−87.86	−105.7	−24.82
10-Demethoxykopsidasinine	−76.25	−62.32	−79.99	−7.03
Pauciflorine a	−106.69	−103.1	−103.19	−76.25
Paprazine	−81.84	−70.23	−87.35	−78.93
Kopsinol	−75.91	−55.94	−97.69	−25.31
Kopsinganol	−107.59	−100.54	−119.99	−52.4
Alpneumine g	−88.68	−81.46	−85.93	−36.89
N-Methylasimilobine-2-O-b-D-glucopyranoside	−103.54	−106.85	−120.85	−103.47
N-Oxide c	−89.91	−97.29	−122.26	−53.85
Pauciflorine b	−90.1	−57.84	−67.57	−50.26
Kopsifoline a	−96.18	−104.64	−114.36	−61.7
Alstonamic acid	−70.89	−85.09	−83.92	−82.8
Isoprostephabyssine	−75.29	−93.55	−83.42	−30.14
Dehatridine	−147.13	−113.85	−103.04	13.7
Jerantinine e	−95.71	−106.3	−109.52	−90.28
Mersifoline a	−86.97	−89.24	−104.91	−57.09
16(S)-10-Metoxi-epi-isositsirikina	−105.02	−105.39	−111.84	−90.46
